# Homozygous deletion of the activin A receptor, type IB gene is associated with an aggressive cancer phenotype in pancreatic cancer

**DOI:** 10.1186/1476-4598-13-126

**Published:** 2014-05-27

**Authors:** Yosuke Togashi, Hiroki Sakamoto, Hidetoshi Hayashi, Masato Terashima, Marco A de Velasco, Yoshihiko Fujita, Yasuo Kodera, Kazuko Sakai, Shuta Tomida, Masayuki Kitano, Akihiko Ito, Masatoshi Kudo, Kazuto Nishio

**Affiliations:** 1 Department of Genome Biology, Kinki University Faculty of Medicine, 377-2 Ohno-higashi, Osaka-Sayama, Osaka 589-8511, Japan; 2 Department of Gastroenterology and Hepatology, Kinki University Faculty of Medicine, Osaka-Sayama, Osaka, Japan; 3 Department of Pathology, Kinki University Faculty of Medicine, Osaka-Sayama, Osaka, Japan

**Keywords:** Pancreatic cancer, Activin signal, Activin A receptor, Type IB, SMAD4

## Abstract

**Background:**

Transforming growth factor, beta (TGFB) signal is considered to be a tumor suppressive pathway based on the frequent genomic deletion of the *SMAD4* gene in pancreatic cancer (PC); however; the role of the activin signal, which also belongs to the TGFB superfamily, remains largely unclear.

**Methods and results:**

We found a homozygous deletion of the activin A receptor, type IB (*ACVR1B*) gene in 2 out of 8 PC cell lines using array-comparative genomic hybridization, and the absence of *ACVR1B* mRNA and protein expression was confirmed in these 2 cell lines. Activin A stimulation inhibited cellular growth and increased the phosphorylation level of SMAD2 and the expression level of p21^CIP1/WAF1^ in the Sui66 cell line (wild-type *ACVR1B* and *SMAD4* genes) but not in the Sui68 cell line (homozygous deletion *of ACVR1B* gene). Stable *ACVR1B*-knockdown using short hairpin RNA cancelled the effects of activin A on the cellular growth of the PC cell lines. In addition, *ACVR1B*-knockdown significantly enhanced the cellular growth and colony formation abilities, compared with controls. In a xenograft study, *ACVR1B*-knockdown resulted in a significantly elevated level of tumorigenesis and a larger tumor volume, compared with the control. Furthermore, in clinical samples, 6 of the 29 PC samples (20.7%) carried a deletion of the *ACVR1B* gene, while 10 of the 29 samples (34.5%) carried a deletion of the *SMAD4* gene. Of note, 5 of the 6 samples with a deletion of the *ACVR1B* gene also had a deletion of the *SMAD4* gene.

**Conclusion:**

We identified a homozygous deletion of the *ACVR1B* gene in PC cell lines and clinical samples and proposed that the deletion of the *ACVR1B* gene may mediate an aggressive cancer phenotype in PC. Our findings provide novel insight into the role of the activin signal in PC.

## Background

Pancreatic cancer (PC) is a devastating disease. Gemcitabine has been the standard therapy for experimental regimens in patients with advanced PC for over a decade, but recently, the overall survival has been significantly prolonged using combination therapies, such as gemcitabine plus erlotinib or a combination of oxaliplatin, irinotecan, fluorouracil and leucovorin (FOLFIRINOX) [1-3]. Despite some recent progress, however, the overall survival rate of patients with PC is still less than 5% [4]. The model explaining the progression of PC is influenced by multiple genetic alterations. During early genetic events, such as activating point mutations in the *K*-*ras* oncogene and the overexpression of the *HER*-*2*/*neu* gene, pancreatic duct lesions show minimal cytological and architectural atypia. The inactivation of the *p16* tumor suppressor gene appears to occur at a later stage, followed by the loss of the *p53*, *SMAD4*, and *BRCA2* tumor suppressor genes [5-8]. For instance, the *HER*-*2*/*neu* gene is not expressed in the epithelium lining of normal pancreatic duct, but it is highly expressed in pancreatic intraepithelial neoplasia [9]. However, two clinical trials assessing anit-HER2 trastuzumab therapy in patients with PC overexpressing HER2 have produced disappointing results [10,11]. Although such recent breakthroughs in the molecular biology of PC have assisted in translational research, creating hope for individualized therapy and better disease management, the inhibition of epidermal growth factor receptor using erlotinib is, to date, the only targeted approach that has been demonstrated to result in a survival [1]. Therefore, further understanding of the molecular biology of PC is needed.

The transforming growth factor, beta (TGFB) receptor II (*TGFBR2*) and *SMAD4* genes are commonly inactivated in several types of cancer, providing evidence that the TGFB signal functions as a tumor suppressor [12,13]. Thirty percent of colorectal cancers are thought to contain a mutation in the *TGFBR2* gene. The human locus 18q21, which encodes the *SMAD2* and *SMAD4* genes, is often mutated or lost completely in several cancers. The loss of the *SMAD4* gene eliminates the classic SMAD2/3/4 heteromeric complexes that have been implicated in a large number of TGFB-dependent transcriptional regulatory complexes. As a result, TGFB-mediated growth inhibition is lost. The *SMAD4* gene is inactivated in 55% of PC tumors, and numerous studies on TGFB signal in PC have been reported. The loss of the *SMAD4* gene is correlated with both a poor prognosis and the development of widespread metastases in patients. The *TGFBR2* gene is also altered in a smaller subset of PC tumors [5-7,14,15]. In addition, pancreatic-specific *TGFBR2* or *SMAD4*-knockout mice with active *K-ras* expression developed PC [16,17]. However, the roles of defects other than those in the *SMAD4* and *TGFBR2* genes in PC remain unclear, and few studies regarding the activin signal, which also belongs to the TGFB superfamily, have been reported [18-20]. Defects in several genes involved in the activin signal pathway have been characterized in several cancers. For instance, two 8-bp polyadenine tracts in the activin A receptor, type IIA (*ACVR2A*) gene were reported to be targets for frameshift mutations in gastrointestinal cancers with microsatellite instability [21]. Similarly, the activin signal induces growth inhibition and apoptosis mainly through SMAD-dependent pathways in many other cancers [22-27]. Thus, the dysregulation of the activin signal is directly involved in carcinogenesis. In contrast, however, a recent study has demonstrated that Nodal/Activin signal is associated with self-renewal and the tumorigenicity of PC stem cells [20]; thus, the role of activin signal in pancreatic carcinogenesis remains controversial. In the present study, we identified a homozygous deletion of the activin A receptor, type IB (*ACVR1B*) gene in PC cell lines using array-comparative genomic hybridization (array-CGH). Furthermore, we investigated the role of this homozygous deletion in PC cell lines and the status of the *ACVR1B* gene in clinical samples of PC.

## Results

### Identification of homozygous deletion of *ACVR1B* gene in PC cell lines

**Figure 1:**
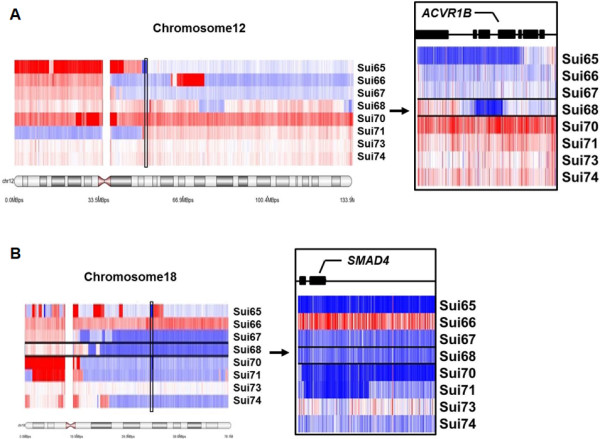
**Array-CGH of PC cell lines.** A gain (>4 copies, red) and a deletion (<0.5 copies, blue) of genomic copy number are shown. **(A)** Array-CGH of chromosome 12. The homozygous deletion of the *ACVR1B* gene was found in the Sui65 and Sui68 cell lines. **(B)** Array-CGH of chromosome 18. The homozygous deletion of the *SMAD4* gene was found in the Sui65, Sui70, and Sui71 cell lines.

The results of an array-CGH demonstrated the homozygous deletion of the *ACVR1B* gene in the Sui65 and Sui68 cell lines (chromosome 12) and the homozygous deletion of the *SMAD4* gene in the Sui65, Sui70, and Sui71 cell lines (chromosome 18) (Figure 1A and B). No deletions of other SMAD genes or other main TGFB and activin receptors, including the *TGFBR1, TGFBR2, ACVR2A, ACVR2B, SMAD2* genes, were found. To examine the *ACVR1B* and *SMAD4* gene copy numbers in the PC cell lines, we used a real-time PCR-based detection method, the TaqMan Copy Number Assay, and the experiment was performed in triplicate. The copy number results are summarized in Table 1. The copy number of the *ACVR1B* gene in the Sui68 cell line was 0 and that in the Sui65 cell line was nearly 0 (0.115 ± 0.025). The copy numbers of the *SMAD4* gene in the Sui65, Sui70, and Sui71 cell lines were all 0. These results were similar to those of the array-CGH.

**Table 1:** Cell line characteristics and the status of ACVR1B and SMAD4

**Cell lines**	**Source**	**Histology**	**ACVR1B**		**SMAD4**	
			**CN**	**Expression**	**CN**	**Expression**
Sui65	Peritoneum	Tubular	0	-	0	-
Sui66	Pancreas	Tubular	1	+	2	++
Sui67	Pancreas	Tubular	1	-	1	+
Sui68	Pancreas	Ad	0	-	1	+
Sui70	Pancreas	Ad	2	+	0	-
Sui71	Liver	Ad	1	+	0	-
Sui73	Pancreas	Tubular	2	+	2	+
Sui74	Pancreas	Tubular	1	+	1	-

ACVR1B, activin receptor A, type IB; Tubular, tubular adenocarcinoma; Ad, adenocarcinoma; CN, gene copy number.

**Legend:** The copy numbers of the *ACVR1B* gene in the Sui65 and Sui68 cell line was 0, and the copy numbers of the *SMAD4* gene in the Sui65, Sui70, and Sui71 cell lines were all 0. *ACVR1B* mRNA was scarcely expressed in the Sui65 and Sui68 cell lines, and *SMAD4* mRNA was also scarcely expressed in the Sui65, Sui70, and Sui71 cell lines. These results were similar to those for the copy number assay.

### mRNA and protein expressions of ACVR1B and SMAD4 in PC cell lines

**Figure 2:**
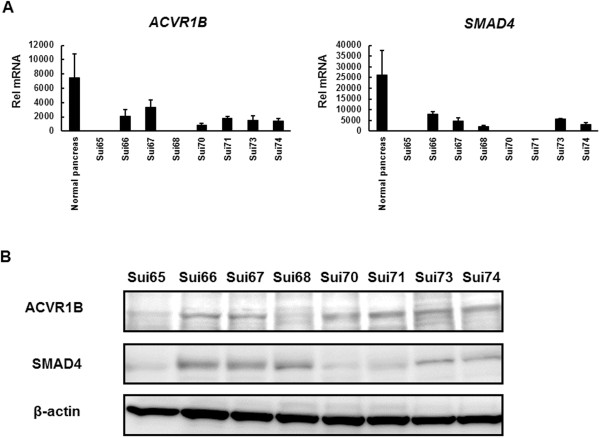
**Expression of *ACVR1B *and *SMAD4 *in PC cell lines. (A)** mRNA expression levels of the *ACVR1B* and *SMAD4* genes in normal pancreatic tissue (RNA from Clontech) and PC cell lines. The expressions were analyzed using real-time RT-PCR. *ACVR1B* mRNA was scarcely expressed in the Sui65 and Sui68 cell lines, and *SMAD4* mRNA was also scarcely expressed in the Sui65, Sui70, and Sui71 cell lines. Rel mRNA, normalized mRNA expression levels (*ACVR1B* or *SMAD4*/*GAPD* × 10^6^); Columns, mean of independent triplicate experiments; Bars, SD. **(B)** Western blot analysis of ACVR1B and SMAD4 in PC cell lines. ACVR1B was scarcely expressed in the Sui65 and Sui68 cell lines. SMAD4 was scarcely expressed in the Sui65, Sui70, and Sui71 cell lines. The findings confirmed the array-comparative genomic hybridization results. β-actin was used as an internal control.

To examine the mRNA expressions of the *ACVR1B* and *SMAD4* genes, we performed real-time reverse transcription PCR (RT-PCR) using samples of normal pancreatic tissue from Clontech and PC cell lines. *ACVR1B* mRNA was scarcely expressed in the Sui65 and Sui68 cell lines, and *SMAD4* mRNA was also scarcely expressed in the Sui65, Sui70, and Sui71 cell lines (Figure 2A). These results were similar to those for the array-CGH and copy number assay (Table 1). Western blot analyses were performed and showed that ACVR1B was scarcely expressed in the Sui65 and Sui68 cell lines and that SMAD4 was scarcely expressed in the Sui65, Sui70, and Sui71 cell lines. The protein expressions of ACVR1B and SMAD4 reflected the mRNA expression levels (Figure 2B).

### Influence of activin A on cellular growth and cell cycle in PC cell lines

**Figure 3:**
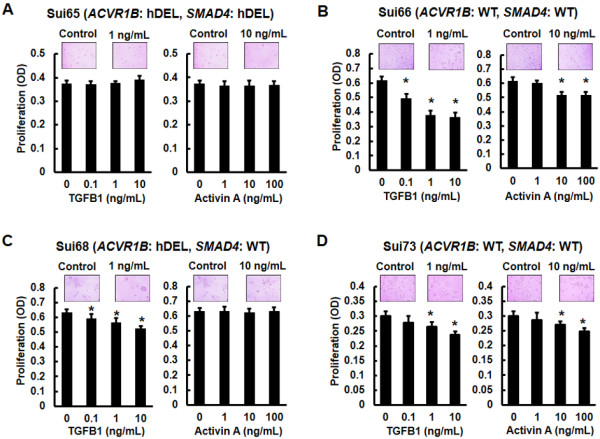
**Influence of TGFB1 and activin A on cellular growth.** The cells were stimulated with the indicated doses of TGFB1 or activin A for 72 hours. Cell proliferation was assayed using an MTT assay. **(A)** Cellular growth of Sui65 cell line (homozygous deletion of *ACVR1B* and *SMAD4* genes). Both TGFB1 (0.1 ng/mL, *P* = 0.56; 1 ng/mL, *P* = 0.51; 10 ng/mL, *P* = 0.69) and activin A (1 ng/mL, *P* = 0.51; 10 ng/mL, *P* = 0.71; 100 ng/mL, *P* = 0.65) did not influence the cellular growth. **(B)** Cellular growth of Sui66 cell line (wild**-**type *ACVR1B* and *SMAD4* genes). Both TGFB1 (0.1 ng/mL, *P* = 0.0049*; 1 ng/mL, *P* = 0.0028*; 10 ng/mL, *P* = 0.0016*) and activin A (1 ng/mL, *P* = 0.051; 10 ng/mL, *P* = 0.010*; 100 ng/mL, *P* = 0.0081*) inhibited cellular growth. **(C)** Cellular growth of Sui68 cell line (homozygous deletion of the *ACVR1B* gene and wild**-**type *SMAD4* gene). TGFB1 inhibited cellular growth (0.1 ng/mL, *P* = 0.011*; 1 ng/mL, *P* = 0.013*; 10 ng/mL, *P* = 0.0039*), but activin A did not influence the cellular growth (1 ng/mL, *P* = 0.65; 10 ng/mL, *P* = 0.93; 100 ng/mL, *P* = 0.82). **(D)** Cellular growth of Sui73 cell line (wild**-**type *ACVR1B* and *SMAD4* genes). As is seen in the Sui66 cell line, both TGFB1 (0.1 ng/mL, *P* = 0.072; 1 ng/mL, *P* = 0.0087*; 10 ng/mL, *P* = 0.0066*) and activin A (1 ng/mL, *P* = 0.21; 10 ng/mL, *P* = 0.0018*; 100 ng/mL, *P* = 0.028*) inhibited cellular growth. hDEL, homozygous deletion; WT, wild-type; Columns, mean of independent triplicate experiments; Bars, SD; *P < 0.05.

To examine the influence of ligands in the PC cell lines, we performed cellular growth assays using the Sui65 (homozygous deletion of *ACVR1B* and *SMAD4* genes), Sui66, Sui73 (wild-type *ACVR1B* and *SMAD4* genes), Sui68 (homozygous deletion of *ACVR1B* gene and wild-type *SMAD4* gene), and Sui70 (wild-type *ACVR1B* and homozygous deletion of *SMAD4* gene) cell lines in the presence of ligands. Based on numerous previous studies and our data on cellular growth inhibition [22,28], we used concentrations of 0.1, 1, or 10 ng/mL of TGFB1 or 1, 10, or 100 ng/mL of activin A. TGFB1 inhibited cellular growth in the Sui66, Sui68, and Sui73 cell lines (Figure 3B, C, and D). Activin A did not influence cellular growth in the Sui65 and Sui68 cell lines (Figure 3A and C), although it inhibited cellular growth in the Sui66 and Sui73 cell lines (Figure 3B and D). In addition, the Sui70 cell line was not influenced by either TGFB1 or activin A (Additional file 1A).

**Figure 4:**
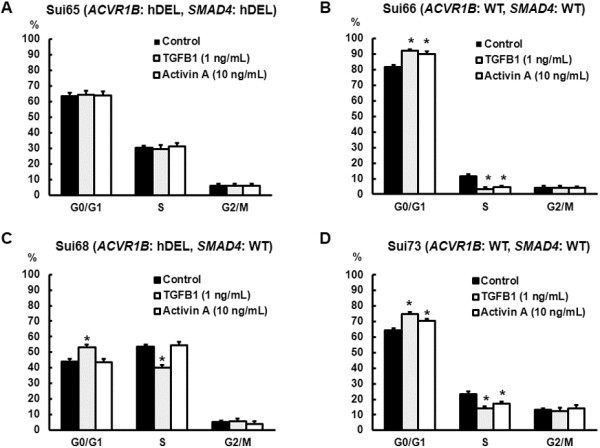
**Influence of TGFB1 and activin A on the cell cycle.** The cell lines were exposed to the ligands (TGFB1, 1 ng/mL; activin A, 10 ng/mL) for 48 hours. The cells were then stained using propidium iodide/RNase Staining Buffer and were analyzed using a flow cytometer. **(A)** Cell cycle distribution of Sui65 cell line (homozygous deletion of *ACVR1B* and *SMAD4* genes). Both TGFB1 and activin A did not influence the cell cycle distribution. **(B)** Cell cycle distribution of Sui66 cell line (wild**-**type *ACVR1B* and *SMAD4* genes). Both TGFB1 and activin A increased the proportion of cells in G0/G1 phase (*P* = 0.0039* and 0.031*, respectively) and decreased the proportion of cells in S phase (*P* = 0.0043* and 0.039*, respectively). **(C)** Cell cycle distribution of Sui68 cell line (homozygous deletion of the *ACVR1B* gene and wild**-**type *SMAD4* gene). TGFB1 increased the proportion of cells in G0/G1 phase (*P* = 0.0016*) and decreased the proportion of cells in S phase (*P* = 0.019*), while activin A did not influence the cell cycle distribution. **(D)** Cell cycle distribution of Sui73 cell line (wild**-**type *ACVR1B* and *SMAD4* genes). As is seen in the Sui66 cell line, both TGFB1 and activin A increased the proportion of cells in G0/G1 phase (*P* = 0.014* and 0.039*, respectively) and decreased the proportion of cells in S phase (*P* = 0.0034* and 0.0021*, respectively). hDEL, homozygous deletion; WT, wild-type; Columns, mean of independent triplicate experiments; Bars, SD; *P < 0.05.

Next, cell cycle distribution analyses were also performed. Both TGFB1 and activin A increased the proportion of cells in the G0/G1 phase and decreased the proportion of cells in the S phase in the Sui66 and Sui73 cell line (Figure 4B and D). In the Sui68 cell line, however, TGFB1 increased the proportion of cells in the G0/G1 phase and decreased the proportion of cells in the S phase, while activin A did not affect the cell cycle distribution (Figure 4C). In the Sui65 cell line, activin A did not affect the cell cycle distribution, either (Figure 4A). These results indicate that activin A inhibits cellular growth and induces G1 phase cell arrest in PC cell lines with wild-type *ACVR1B*, while activin A does not inhibit cellular growth and does not influence the cell cycle in cell lines with a homozygous deletion of the *ACVR1B* gene.

### Effect of activin A on SMAD2 phosphorylation and p21 induction in PC cell lines

**Figure 5:**
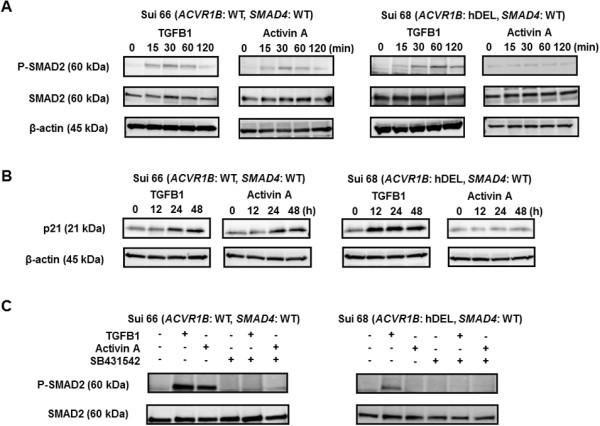
**Western blot analyses using Sui66 and Sui68 cell lines.** The cell lines were treated with or without 2 μM of SB431542 for 30 min, then stimulated with TGFB1 (1 ng/mL) or activin A (10 ng/mL). β-actin was used as an internal control. **(A)** Phosphorylation of SMAD2 in Sui66 (wild**-**type *ACVR1B* and *SMAD4* genes) and Sui68 (homozygous deletion of the *ACVR1B* gene and wild**-**type *SMAD4* gene) cell lines without SB431542. Both TGFB1 and activin A increased the phosphorylation levels of SMAD2 in Sui66 cell line. TGFB1 increased the phosphorylation level of SMAD2, but activin A did not influence phosphorylation in Sui68 cell line. **(B)** Expression of p21 in Sui66 and Sui68 cell lines without SB431542. The expression of p21 was evaluated in whole-cell lysates. Although p21 expression was increased by both TGFB1 and activin A in Sui66 cell line, its expression was increased only by TGFB1 in Sui68 cell line. **(C)** Phosphorylation of SMAD2 in Sui66 and Sui68 cell lines with or without SB431542. The cell line was stimulated by TGFB1 or activin A for 1 hour. The phosphorylation levels of SMAD2 increased in response to both TGFB1 and activin A but were cancelled by SB431542 in Sui66 cell line. The phosphorylation level of SMAD2 increased only in response to TGFB1 and was cancelled by SB431542 in Sui68 cell line.

Activin A inhibited the cellular growth of cell lines with wild-type *ACVR1B* and *SMAD4* genes; therefore, we examined the downstream signal under TGFB1 or activin A stimulation. Based on numerous previous studies and our data on cellular growth inhibition [22,28], we used 1 ng/mL of TGFB1 or 10 ng/mL of activin A. The time points were also decided based on the previous studies [22,28]. In the Sui66 cell line (wild-type *ACVR1B* and *SMAD4* genes), both TGFB1 and activin A increased the phosphorylation levels of SMAD2 (Figure 5A); these effects were cancelled by the ACVR1B/TGFBR1/ACVR1C-specific inhibitor SB431542 (Figure 5C). In the Sui68 cell line (homozygous deletion of *ACVR1B* gene and wild-type *SMAD4* gene), TGFB1, but not activin A, increased the phosphorylation levels of SMAD2 (Figure 5A); these effects were cancelled by SB431542 (Figure 5C). These results suggest that activin A activates the SMAD signal in a manner similar to TGFB1 in PC cell lines with the wild-type *ACVR1B* gene, but does not activate in PC cell lines with the homozygous deletion of the *ACVR1B* gene.

Next, we evaluated the expression levels of p21^CIP1/WAF1^. p21^CIP1/WAF1^ is a major cdk inhibitor and is a hallmark of the cytostatic role of the TGFB signal pathway [29]. TGFB and activin A are known to increase p21 expression [22,28]. The expression of p21 was evaluated in whole-cell lysates. p21 expression was increased by both TGFB1 and activin A in the Sui66 cell line. In the Sui68 cell line, however, its expression was increased only by TGFB1 (Figure 5B). Therefore, we speculated that p21 may have a role in activin A-mediated growth inhibition and cell-cycle progression.

To evaluate the effect of activin A on SMAD4-independent pathways, the phosphorylation of ERK1/2 and AKT, which are representative signals of SMAD4-independent pathways, was investigated in the Sui70 cell line (wild-type *ACVR1B* and homozygous deletion of *SMAD4* genes). The phosphorylation was not changed by activin A (Additional file 1B). Particularly, both AKT and ERK1/2 were phosphorylated before the stimulations. The expression of p21 also remained unchanged.

### Enhanced cellular growth and colony formation, but no response to activin A, of Sui66/shACVR1B and Sui73/shACVR1B cell lines

**Figure 6:**
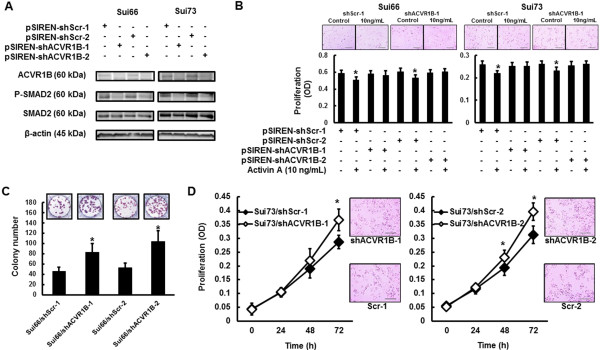
**Role of *ACVR1B *gene in colony formation and cellular growth.** To evaluate the role of the *ACVR1B* gene, we used stable *ACVR1B*-knockdown cell lines (shACVR1B) or control (shScr). **(A**) Western blot analyses after activin A stimulation (10 ng/mL). In contrast to Sui66/shScr and Sui73/shScr cell lines, ACVR1B was not expressed in Sui66/shACVR1B and Sui73/shACVR1B cell lines. SMAD2 was not phosphorylated by activin A in Sui66/shACVR1B and Sui73/shACVR1B cell lines. β-actin was used as an internal control. **(B)** Influence of activin A on cellular growth. The cell lines were stimulated with or without activin A (10 ng/mL) for 72 hours. Cell proliferation was assayed using an MTT assay. Although activin A inhibited cellular growth in Sui66/shScr and Sui73/shScr cell lines (Sui66/shScr-1; *P* = 0.0014*, Sui66/shScr-2; *P* = 0.0032*, Sui73/shScr-1; *P* = 0.0085*, and Sui73/shScr-2, *P* = 0.0053*, respectively), it did not influence the cellular growth in Sui66/shACVR1B and Sui73/shACVR1B cell lines (Sui66/shACVR1B-1; *P* = 0.81, Sui66/shACVR1B-2; *P* = 0.73, Sui73/shACVR1B-1; *P* = 0.90, and Sui73/shACVR1B-2; *P* = 0.87, respectively). **(C)** Colony formation of Sui66**-**transfectant cell lines. The colony formations in Sui66/shACVR1B cell lines were enhanced, compared with that in the control cell lines (Scr-1, 46.11 ± 8.00 vs. ACVR1B-1, 83.00 ± 16.75, *P* = 0.026* and Scr-2, 52.55 ± 9.24 vs. ACVR1B-2, 103.70 ± 21.00, *P* = 0.017*). **(D)** Cellular growth of Sui73**-**transfectant cell lines. The cellular growth was evaluated using an MTT assay. The cellular growths in Sui73/shACVR1B cell lines were enhanced, compared with Sui73/shScr (shScr-1 vs. shACVR1B-1, 0 h, *P* = 081; 24 h, *P* = 0.88; 48 h, *P* = 0.063; 72 h, *P* = 0.013*, and shScr-2 vs. shACVR1B-2, 0 h, *P* = 040; 24 h, *P* = 0.45; 48 h, *P* = 0.040*; 72 h, *P* = 0.014*). Columns, mean of independent triplicate experiments; Lines, mean of independent triplicate experiments; Bars, SD; *P < 0.05.

To evaluate the role of the *ACVR1B* gene, we examined the colony formation and the cellular growth of stable *ACVR1B*-knockdown cell lines (Sui66/shACVR1B-1, Sui66/shACVR1B-2, Sui73/shACVR1B-1, and Sui73/shACVR1B-2) or control cell lines (Sui66/shScr-1, Sui66/shScr-2, Sui73/shScr-1, and Sui73/shScr-2) (Figure 6A). Activin A did not increase the phosphorylation level of SMAD2 in the Sui66/shACVR1B-1, Sui66/shACVR1B-2, Sui73/shACVR1B-1, or Sui73/shACVR1B-2 cell lines (Figure 6A). Although activin A inhibited the cellular growth of the Sui66/shScr-1, Sui66/shScr-2, Sui73/shScr-1, and Sui73/shScr-2 cell lines, it did not influence the cellular growth of the Sui66/shACVR1B-1, Sui66/shACVR1B-2, Sui73/shACVR1B-1, or Sui73/shACVR1B-2 cell lines (Figure 6B). The colony formation and cellular growth of the Sui66/shACVR1B-1, Sui66/shACVR1B-2, Sui73/shACVR1B-1, and Sui73/shACVR1B-2 cell lines were also enhanced, compared with the controls (Figure 6C and D). These results indicate that the *ACVR1B* gene is involved in tumorigenicity and cellular growth.

### Enhanced *in vivo* tumorigenicity and tumor growth of stable *ACVR1B*-knockdown cell lines

**Figure 7:**
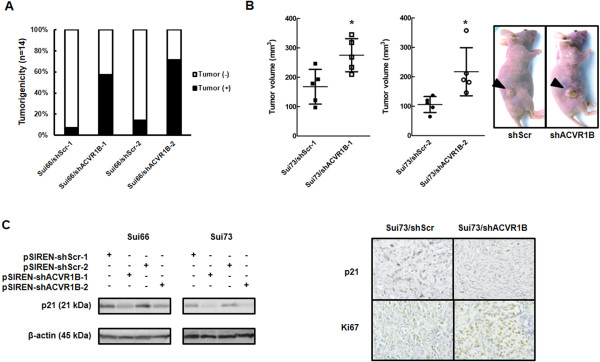
**Xenograft study in Sui66- and Sui73-transfectant cell lines. (A)** Tumorigenesis *in vivo*. To evaluate tumorigenicity *in vivo*, a suspension of 5 × 10^6^ Sui66-transfectant cells (in 50 μL PBS) were subcutaneously inoculated into both flanks of nude mice (n = 7). Tumor formation was assessed every 2 or 3 days. Sui66/sh-ACVR1B exhibited a significantly elevated level of tumorigenesis *in vivo* (Scr-1 1/14 vs. ACVR1B-1 8/14, *P* = 0.013* and Scr-2 2/14 vs. ACVR1B-2 10/14, *P* = 0.0063*). **(B)** Tumor growth *in vivo*. To assess the tumor growth *in vivo*, a suspension of 5 × 10^6^ Sui73-transfectant cell lines (in 50 μL PBS) with 50 μL of Matrigel were subcutaneously inoculated into the right flank of nude mice (n = 5). The tumor volume was assessed every 2 or 3 days. Sui73/shACVR1B exhibited a significantly elevated tumor volume (Sui73/Scr-1, 167.7 ± 59.1 mm^3^ vs. Sui73/ACVR1B-1, 275.0 ± 56.3 mm^3^; *P* = 0.018* on day 36 and Sui73Scr-2, 105.7 ± 27.2 mm^3^ vs. Sui73/ACVR1B-2, 217.3 ± 81.8 mm^3^; *P* = 0.020* on day 29, respectively). Lines, mean of 5 tumors; Error bars, SD; *P < 0.05. **(C)** Western blot analyses of the tumors and immunostaining. The expressions of p21 were clearly elevated in cancer cells in shScr-inoculated tumors, compared with the expression levels in shACVR1B cells. The expressions of Ki67 were also clearly elevated in the cancer cells in the shACVR1B-inculated tumors. β-actin was used as an internal control.

We evaluated the *in vivo* tumorigenicity of Sui66-transfectant cell lines and the tumor growth of Sui73-transfectant cell lines. Sui66/shACVR1B exhibited a significantly elevated level of tumorigenesis (Sui66/shScr-1 1/14 vs. Sui66/shACVR1B-1 8/14, *P* = 0.013 and Scr-2 2/14 vs. ACVR1B-2 10/14, *P* = 0.0063*), and Sui73/shACVR1B exhibited a larger tumor volume than Sui73/shScr (Sui73/Scr-1, 167.7 ± 59.1 mm^3^ vs. Sui73/ACVR1B-1, 275.0 ± 56.3 mm^3^; *P* = 0.018* on day 36 and Sui73Scr-2, 105.7 ± 27.2 mm^3^ vs. Sui73/ACVR1B-2, 217.3 ± 81.8 mm^3^; *P* = 0.020* on day 29, respectively). (Figure 7A and B). There was no significant difference in body weight (Sui73/Scr-1, 24.3 ± 1.0 g vs. Sui73/ACVR1B-1, 23.24 ± 1.5 g; *P* = 0.22 on day 36 and Sui73/Scr-2, 21.6 ± 1.3 g vs. Sui73/ACVR1B-2, 21.1 ± 1.8 g; *P* = 0.61 on day 29, respectively). According to western blot analyses of the tumors and immunostaining, the expressions of p21 were clearly elevated in the cancer cells in the shScr-inoculated tumors, compared with the expression levels in the shACVR1B cells (Figure 7C). In addition, the expressions of Ki67 were clearly elevated in the cancer cells in the shACVR1B-inoculated tumors (Figure 7C). These results indicate that the *ACVR1B* gene is involved in tumorigenicity and tumor growth and that it downregulated the expression level of p21 in cancer cells *in vivo*, similar to its effect *in vitro*.

### Suppressed *in vitro* cellular growth and colony formation, and *in vivo* tumorigenicity of p21-overexpressed Sui68 cell line

**Figure 8:**
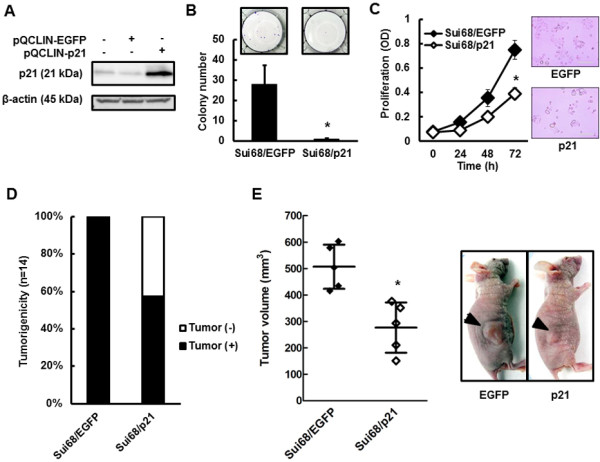
**Role of *p21 *gene in colony formation and cellular growth *in vitro *and tumorigenicity *in vivo*.** To see if p21 expression reverses the phenotype for *ACVR1B* gene homozygous deletion, we created a *p21*-overexpressed Sui68 cell line (homozygous deletion of *ACVR1B* gene and wild-type *SMAD4* gene). **(A)** Western blot analyses. The overexpression of p21 was confirmed using western blot analyses in the Sui68/p21 cell line. β-actin was used as an internal control. **(B)** Colony formation of Sui68**-**transfectant cell lines. The colony formation in the Sui68/p21 cell line was suppressed, compared with that in the control cell line (EGFP, 28.0 ± 9.17 vs. p21, 0.89 ± 0.19, *P* = 0.035*). Columns, mean of independent triplicate experiments; Bars, SD; *P < 0.05. **(C)** Cellular growth of Sui68**-**transfectant cell lines. The cellular growth was evaluated using an MTT assay. Cellular growth in the Sui68/p21 cell line was suppressed, compared with Sui68/EGFP (0 h, *P* = 065; 24 h, *P* = 0.074; 48 h, *P* = 0.053; 72 h, *P* = 0.030*). Lines, mean of independent triplicate experiments; Bars, SD; *P < 0.05. **(D)** Tumorigenesis *in vivo*. To evaluate tumorigenicity *in vivo*, a suspension of 5 × 10^6^ Sui68-transfectant cells (in 50 μL PBS) were subcutaneously inoculated into both flanks of nude mice (n = 7). Sui68/EGFP exhibited a significantly elevated level of tumorigenesis (Sui68/EGFP 14/14 vs. Sui68/p21 8/14, *P* = 0.016). **(E)** Tumor growth *in vivo.* To evaluate the tumor growth, a suspension of 5× 10^6^ Sui68-transfectant cells (in 50 μL PBS) with 50 μL of Matrigel were subcutaneously inoculated into the right flanks of nude mice (n = 5). Sui68/EGFP exhibited a larger tumor volume than Sui68/p21 on day 15 (Sui68/EGFP, 507.0 ± 83.5 mm^3^ vs. Sui68/p21, 276.5 ± 95.0 mm^3^; *P* = 0.0036*). Lines, mean of five tumors; Bars, SD; *P < 0.05.

To determine whether p21 expression reverses the phenotype of the *ACVR1B* gene homozygous deletion, we created a p21-overexpressed Sui68 cell line (homozygous deletion of *ACVR1B* gene and wild-type *SMAD4* gene) (Figure 8A). The colony formation and cellular growth of the Sui68/p21 cell line were greatly suppressed, compared with the controls (Figure 8B and C). In addition, Sui68/EGFP exhibited a significantly elevated level of tumorigenesis (Sui68/EGFP 14/14 vs. Sui68/p21 8/14, *P* = 0.016), and Sui68/EGFP exhibited a larger tumor volume than Sui68/p21 on day 15 (Sui68/EGFP, 507.0 ± 83.5 mm^3^ vs. Sui68/p21, 276.5 ± 95.0 mm^3^; *P* = 0.0036*) (Figure 8D). No significant difference in body weight was seen on day 15 (Sui68/EGFP, 20.3 ± 0.8 g vs. Sui68/p21, 20.2 ± 1.5 g; *P* = 0.94). These results suggest that p21 expression reverses the phenotype arising from the homozygous deletion of the *ACVR1B* gene.

### ACVR1B and SMAD4 gene copy numbers in clinical samples of PC

**Figure 9:**
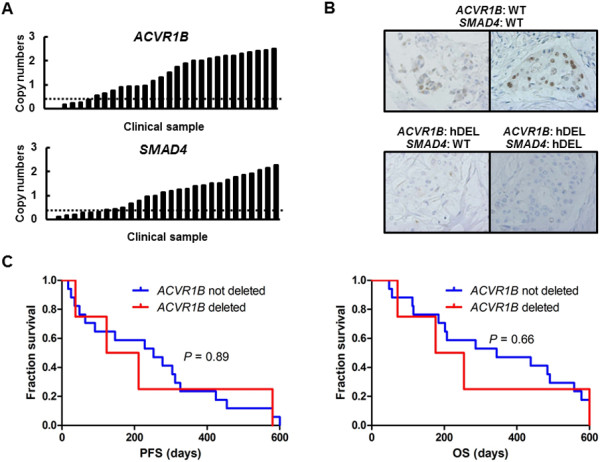
**Copy numbers of *ACVR1B *and *SMAD4 *genes, immunostaining of p21, and Kaplan-Meier curves for PFS and OS in PC clinical samples. (A)** The copy numbers were analyzed using TaqMan copy number assays. Copy number of the *ACVR1B* gene. Six samples (6/29, 20.7%) had a copy number of less than 0.5 (deletion). Copy number of the *SMAD4* gene. Ten samples (10/29, 34.5%) had a copy number of less than 0.5 (deletion). **(B)** Immunostaining of p21. The expressions of p21 were clearly elevated in the cancer cells of patients with wild-type *ACVR1B* and *SMAD4* genes, compared with the expression levels in those of patients with a homozygous deletion of the *ACVR1B* gene. WT, wild-type; hDEL, homozygous deletion. **(C)** Kaplan-Meier curves for PFS and OS. Among 21 patients who received chemotherapy, no significant differences in PFS or OS were seen between the patients without a homozygous deletion of the *ACVR1B* gene and those with such a deletion (median PFS, 252 days vs. 167 days, P = 0.89, and median OS, 344 days vs. 215 days, P = 0.66, respectively).

To examine the *ACVR1B* and *SMAD4* gene copy numbers in PC clinical samples, we performed a TaqMan Copy Number Assay. The *ACVR1B* gene copy numbers in 6 samples (6/29, 20.7%) were less than 0.5 (Figure 9A), while the *SMAD4* gene copy numbers in 10 samples (10/29, 34.5%) were less than 0.5 (Figure 9A). The association between the patient characteristics and the *ACVR1B* gene status is summarized in Table 2. Interestingly, 5 of the 6 samples with a deletion of the *ACVR1B* gene also had a deletion of the *SMAD4* gene (*P* = 0.011), but no significant differences in the other patient characteristics were observed between the two groups. According to the immunostaining results, the expressions of p21 were clearly elevated in the cancer cells of patients with wild-type *ACVR1B* and *SMAD4* genes, compared with the expression levels in patients with the homozygous deletion of the *ACVR1B* gene (Figure 9B). Twenty-one patients with a good performance status received chemotherapy (gemcitabine, n = 12; gemcitabine/S1, n = 5; S1, n = 4) at Kinki University Hospital. These regimens were commonly used in Japan before the availability of erlotinib or FOLFIRINOX. Among these patients, no significant differences in progression-free survival (PFS) or overall survival (OS) were seen between the two groups (Figure 9C and Table 2).

**Table 2:** **Patient characteristics and the association with *ACVR1B *** **gene status**

**Patients characteristics**	***ACVR1B *** **gene**		***P***
	**Not deletion (n = 23)**	**Deletion (n = 6)**	
Age			
<70 years	15	3	0.65
≥ 70 years	8	3	
Gender			
Male	12	3	1.00
Female	11	3	
Primary size			
<2 cm	18	5	1.00
≥2 cm	5	1	
Lymph node metastasis			
Negative	4	0	0.55
Positive	19	6	
Distant metastasis			
Negative	10	4	0.39
Positive	13	2	
*SMAD4* gene status			
Not deletion	18	1	0.011*
Deletion	5	5	
Treatment			
Best supportive care	4	2	0.59
Chemotherapy	17	4	
Unknown	2	0	
Response to chemotherapy			
PR	7	1	1.00
SD or PD	10	3	
Median PFS (days)	252	167	0.89
Median OS (days)	344	215	0.66

ACVR1B, activin receptor A, type IB; PR, partial response; SD, stable disease; PD, progressive disease; PFS, progression-free survival; OS, overall survival. PFS and OS were analyzed by log-rank test and the others by Fisher exact test.

**Legend:** Six patients (6/29, 20.7%) had a deletion of *ACVR1B* gene, 10 patients (10/29, 34.5%) had a deletion of *SMAD4* gene. Five of the 6 patients with a deletion of the *ACVR1B* gene also had a deletion of the *SMAD4* gene (*P* = 0.011), but no significant differences in the other patient characteristics were observed between the two groups. Among 21 patients who received chemotherapy, no significant differences in PFS or OS were seen between the two groups.

## Discussion

In this study, we identified a homozygous deletion of the *ACVR1B* gene in PC cell lines and clinical samples. Activin A inhibited cellular growth in the cell lines with wild-type *ACVR1B* and *SMAD4* genes, and *ACVR1B*-knockdown enhanced cellular growth and colony formation *in vitro* as well as tumor growth and tumorigenicity *in vivo*. These results suggest that the activin signal has a tumor suppressive role in PC and that the deletion of the *ACVR1B* gene mediates an aggressive cancer phenotype relative to PC carrying the wild-type *ACVR1B* gene.

Similar to our study, several articles have shown an anti-tumorigenic effect of the activin signal. Activin A induces growth inhibition and apoptosis mainly through SMAD-dependent pathways in many other cancers, such as gall bladder cancer, prostate cancer, neuroblastoma, breast cancer, ovarian cancer, and colon cancer [22-27]. In our study, activin A inhibited cellular growth and induced G1 phase cell arrest in a PC cell line with wild-type *ACVR1B* and *SMAD4* genes via the phosphorylation of SMAD2 and the expression of p21, while the cellular growth of a cell line with the homozygous deletion of the *ACVR1B* gene was not inhibited by activin A. In addition, the cellular inhibitory effect of activin A and the activin-induced phosphorylation of SMAD2 were cancelled by *ACVR1B*-knockdown in cell lines with wild-type *ACVR1B* and *SMAD4* genes. *In vivo*, *ACVR1B*-knockdown also enhanced the tumorigenicity and tumor growth. The Sui68 cell line (homozygous deletion of the *ACVR1B* gene and the wild-type *SMAD4* gene) was capable of generating sufficient tumors, suggesting that the deletion of the *ACVR1B* gene contributes to tumorigenicity even in the presence of the wild-type *SMAD4* gene. In addition, the *in vitro* colony formation and cellular growth and the *in vivo* tumorigenicity of the Sui68 cell line were greatly inhibited by *p21*-overexpression. Thus, the anti-tumorigenic effect of the activin signal via the SMAD pathways and p21 was lost by *ACVR1B-*knockdown, which was related to an aggressive phenotype of PC.

Interestingly, another article and the present study both demonstrated that the inactivation of the *ACVR1B* gene is frequently complicated by the inactivation of the *SMAD4* gene [19]. The TGFB and activin signals have non-SMAD pathways, and the ERK/MAPK signal and the PI3K/AKT signal are representative signals that are associated with cellular growth and survival [30]. In the Sui70 cell line (wild-type *ACVR1B* gene and the homozygous deletion of the *SMAD4* gene), however, activin A did not influence the cellular growth, and neither the phosphorylation of ERK1/2 nor that of AKT was enhanced. Approximately 95% of PC, including the Sui70 cell line has a *K-ras* mutation [5-8], and both ERK1/2 and AKT are phosphorylated in the Sui70 cell line because of this mutation [31]. Therefore, non-SMAD pathways may have little effect on the aggressiveness of PC carrying the wild-type *ACVR1B* gene and a homozygous deletion of the *SMAD4* gene. In addition, some other reports have suggested that the inactivation of a TGFB receptor is not mutually exclusive with that of the *SMAD4* gene, since both members are known to be genetically inactivated in some tumors [32,33]. Therefore, these findings would fit with a combined input model, which could explain the observed coexistence of the genetic inactivations of these genes.

Pancreatic-specific *TGFBR2* or *SMAD4*-knockout mice with active *K-ras* expression reportedly developed PC [16,17]. However, systemic *ACVR1B*-knockout mice do not survive beyond embryonic day 9.5 [34], and pancreatic-specific *ACVR1B*-knockout mice have not been previously studied. Considering the tumor suppressive role of the *ACVR1B* gene, the development of PC in pancreatic-specific *ACVR1B*-knockout mice seems reasonable. In contrast to our results, however, a recent study has demonstrated that Nodal/Activin signal is associated with self-renewal and the tumorigenicity of PC stem cells [20]. Therefore, to investigate these findings, further research is required.

Since the clinical DNA samples were obtained using needle biopsies, the inclusion of some normal pancreas tissue was unavoidable. Thus, the copy number data does not exactly reflect that for cancer tissue. In addition, the number of clinical samples was very small. Therefore, this cohort has many limitations. No significant differences in the PFS or OS were observed between the patients with *ACVR1B* gene deletions and those without. To confirm the clinical importance of the *ACVR1B* gene deletion, larger studies including more precise genome evaluations are needed.

## Conclusion

We identified the homozygous deletion of the *ACVR1B* gene in PC cell lines and clinical samples. Our experimental findings indicate that the activin signal has a tumor suppressive role and that the deletion of the *ACVR1B* gene may mediate an aggressive cancer phenotype in PC.

## Materials and methods

### Cell culture, ligands, and reagents

Human PC cell lines (Sui65, Sui66, Sui67, Sui68, Sui70, Sui71, Sui73, and Sui74) were maintained in RPMI-1640 medium (Sigma-Aldrich, St. Louis, MO) with 10% FBS (GIBCO BRL, Grand Island, NY) (Table 1) [31]. The cell lines were maintained in a 5% CO_2_-humidified atmosphere at 37˚C.

TGFB1 and activin A were both purchased from R&D Systems (Minneapolis, MN). The ACVR1B/TGFBR1/ACVR1C-specific inhibitor SB431542 was purchased from Sigma-Aldrich.

### Array-based comparative genomic hybridization

The Genome-wide Human SNP Array 6.0 (Affymetrix, Santa Clara, CA) was used to perform array-CGH on genomic DNA from each of the PC cell lines as described previously [35]. The GeneChip Human Mapping 250 K Nsp Array (Affymetrix) was used to perform array-CGH on genomic DNA from each of the cell lines. A total of 250 ng of genomic DNA was digested with Nsp I (250 K) or both Nsp I and Sty I in independent parallel reactions (SNP6.0), subjected to restriction enzymes, ligated to the adaptor, and amplified using PCR with a universal primer and TITANIUM Taq DNA Polymerase (Clontech, Palo Alto, CA). The PCR products were then quantified, fragmented, end-labeled, and hybridized onto a GeneChip Human Mapping 250 K Nsp Array or a Genome-wide Human SNP6.0 Array. After washing and staining in Fluidics Station 450 (Affymetrix), the arrays were scanned to generate CEL files using the GeneChip Scanner 3000 and GeneChip Operating Software, ver.1.4.

### Copy number assay for ACVR1B and SMAD4 genes

The copy numbers for *ACVR1B* and *SMAD4* genes were determined using commercially available and pre-designed TaqMan Copy Number Assays (Applied Biosystems, Foster City, CA) as described previously [36]. The primer IDs used for the *ACVR1B* and *SMAD4* genes were Hs06931689_cn (intron 1) and Hs07120826_cn (intron 1), respectively. The *TERT* locus was used for the internal reference copy number. Human Genomic DNA (TaKaRa) was used as a normal control. Real-time genomic PCR was performed in a total volume of 20 μL in each well, which contained 10 μL of TaqMan genotyping master mix and 20 ng of genomic DNA and each primer. The PCR conditions were 95°C for 10 min and 40 cycles of 95°C for 15 sec and 60°C for 1 min; the resulting products were detected using the ABI PRISM 7900 HT Sequence Detection System (Applied Biosystems). Data were analyzed using SDS 2.2 software and CopyCaller software (Applied Biosystems). Samples with a gene copy number of less than 0.5 were defined as having a copy number of 0 (deletion of the gene), while those with a gene copy number of 0.5 or more but less than 1.5 were defined as having a copy number of 1 and those with a gene copy number of 1.5 or more but less than 2.5 were defined as having a copy number of 2.

### Real-time RT-PCR

One microgram of total RNA from each of the PC cell lines and normal pancreas tissue purchased from Clontech were converted to cDNA using the GeneAmp RNA-PCR kit (Applied Biosystems). Real-time PCR was performed using the Applied Biosystems 7900 HT Fast Real-time PCR System (Applied Biosystems), as described previously [28] under the following conditions: 95°C for 5 min, 50 cycles of 95°C for 10 sec, and 60°C for 1 min. Glyceraldehyde 3 phosphate dehydrogenase (*GAPD*, NM_002046) was used to normalize the expression levels in subsequent quantitative analyses. To amplify the target genes, the following primers were used: *ACVR1B*-F, CAGCAGAACCTTGGCGGTTTA; *ACVR1B*-R, GTTGGCAGATCCCAGAGGCTAC; *SMAD4*-F, CAGCTATGCCAGAAGCCAGA; *SMAD4*-R, GAACTCCTGGGACTTTCAACTGAC; *GAPD*-F, GCACCGTCAAGGCTGAGAAC; *GAPD*-R, ATGGTGGTGAAGACGCCAGT. The experiment was performed in triplicate.

### Plasmid construction, viral production, and stable transfectants

A short hairpin RNA (shRNA)-targeting *ACVR1B* gene was constructed using oligonucleotides encoding small interfering RNA directed against the *ACVR1B* gene and a non-specific target as follows: GAATTGCTCATCGAGACTT and GGCTTGTTTCTGACTATCA for ACVR1B shRNA (shRNA ACVR1B-1 and shRNA ACVR1B-2, respectively), and ACTTGGTTCGCGTATCAAA and CCATATTGCGCGTTGATTT for control shRNA (shRNA scramble-1 and shRNA scramble-2, respectively). The method was described previously [28]. Briefly, the oligonucleotides were cloned into an RNAi-Ready pSIREN-RetroQZsGreen vector (Clontech). A pVSV-G vector (Clontech) for the constitution of the viral envelope and the RNAi-Ready pSIREN-RetroQZsGreen constructs were cotransfected into gpIRES-293 cells using FuGENE6 transfection reagent (Roche Diagnostics, Basel, Switzerland). After 48 hours of transfection, the culture medium was collected and the viral particles were concentrated by centrifugation at 15,000 × *g* for 3 hours at 4°C. The viral pellet was then resuspended in fresh RPMI-1640 medium. The titer of the viral vector was calculated by counting the green-positive cells that were infected by serial dilutions of virus-containing media, and the multiplicity of infection was then determined. The viral vectors were designated as pSIREN-shACVR1B-1, pSIREN-shACVR1B-2, pSIREN-shScr-1, and pSIREN-shScr-2. The stable transfectants expressing shRNA ACVR1B-1, shRNA ACVR1B-2, shRNA scramble-1 or shRNA scramble-2 in the Sui66 and Sui73 cell lines were designated as Sui66/shACVR1B (Sui66/shACVR1B-1 and Sui66/shACVR1B-2), Sui66/shScr (Sui66/shScr-1 and Sui66/shScr-2), Sui73/shACVR1B (Sui73/shACVR1B-1 and Sui73/shACVR1B-2), and Sui73/shScr (Sui73/shScr-1 and Sui73/shScr-2), respectively.

A full-length cDNA fragment encoding the human *p21* gene was introduced into a pQCLIN retroviral vector (Clontech) together with enhanced green fluorescent protein (EGFP) following the internal ribosome entry site sequence (IRES) to monitor the expression of the inserts indirectly. The methods used for viral production and the stable transfectant were described above. The vectors and stable viral transfectant Sui68 cell line was designated as pQCLIN-EGFP, pQLCIN-p21, Sui68/EGFP and Sui68/p21, respectively.

### Antibody

A goat antibody specific for ACVR1B was obtained from R&D Systems. Rabbit antibodies specific for SMAD2, phospho-SMAD2, SMAD4, AKT, phospho-AKT, ERK1/2, phospho-ERK1/2, p21, and β-actin were obtained from Cell Signaling (Beverly, MA).

### Western blot analysis

A western blot analysis was performed as described previously [28]. Briefly, subconfluent cells were washed with cold phosphate-buffered saline (PBS) and harvested with Lysis A buffer containing 1% Triton X-100, 20 mM Tris–HCl (pH7.0), 5 mM EDTA, 50 mM sodium chloride, 10 mM sodium pyrophosphate, 50 mM sodium fluoride, 1 mM sodium orthovanadate, and a protease inhibitor mix, Complete™ (Roche Diagnostics). Whole-cell lysates were separated using a 5%-20% SDS-PAGE and were blotted onto a polyvinylidene fluoride membrane. After blocking with 3% bovine serum albumin in a TBS buffer (pH8.0) with 0.1% Tween-20, the membrane was probed with primary antibody. After rinsing twice with TBS buffer, the membrane was incubated with horseradish peroxidase-conjugated secondary antibody and washed, followed by visualization using an ECL detection system (GE Healthcare, Buckinghamshire, United Kingdom) and LAS-3000 (Fujifilm, Tokyo, Japan). When the influence of the ligands was evaluated, the cultured medium was replaced with 1% FBS medium 6 hours before exposure to the ligands.

### Cellular growth assay

The Sui66-, Sui68-, and Sui73-transfectant cell lines were incubated on 96-well plates at a density of 2,000/well with 200 μL of cultured medium at 37°C in 5% CO_2_. After 24, 48, or 72 hours of incubation, 20 μL of MTT [3-(4,5-dimethyl-thiazoyl-2-yl)2,5-diphenyltetrazolium bromide] solution (Sigma-Aldrich) was added and the culture medium was discarded; the wells were then filled with DMSO. The absorbance of the cultures at 570 nm was measured using VERSAmax (Japan Molecular Devices, Tokyo, Japan). To evaluate growth in the presence of ligands, we also used an MTT assay. The cell lines (2,000/well) were transferred to 96-well plates and cultured using 1% FBS medium for 24 hours at 37°C. Then, the ligands (TGFB1: 0, 0.1, 1, and 10 ng/mL; activin A: 0, 1, 10, and 100 ng/mL) were added and the incubation was further continued for 72 hours at 37°C using 1% FBS medium. The average O.D. values of the 6 wells were used for a single experiment, and the experiment was performed in triplicate.

### Colony formation assay

Sui66- and Sui73-transfectant cell lines were seeded into 6-well plates at a density of 200 cells/well, and Sui68- transfectant cell lines were seeded into 6-well plates at a density of 500 cells/well. After 2 weeks, the cells were washed with PBS and fixed with 4% paraformaldehyde for 10 min and then stained with 0.1% crystal violet for 15 min; the colonies were then counted under a light microscope. The experiment was performed in triplicate.

### Cell cycle distribution analysis

The cell cycle analyses were performed as described previously [37]. Briefly, cell lines were seeded into 6-cm dishes of 2 × 10^5^ cells and cultured using 1% FBS medium for 24 hours at 37°C. Then, the ligands (TGFB1, 1 ng/mL; activin A, 10 ng/mL) were added, and the incubation was further continued for 48 hours at 37°C using 1% FBS medium. Cells were harvested by trypsinization, washed twice with PBS, and fixed with cold 70% ethanol at 4°C for 30 min. Then, the cells were washed twice with PBS and stained using propidium iodide/RNase Staining Buffer (BD Biosciences, San Jose, CA) at room temperature for 15 min. The cells were analyzed using a flow cytometer (BD FACSCalibur™, BD Biosciences), and the cell cycle analysis was performed using ModFit LT software. The experiment was performed in triplicate.

### Xenograft studies

Nude mice (BALB/c nu/nu; 6-week-old females; CLEA Japan, Tokyo, Japan) were used for the *in vivo* studies and were cared for in accordance with the recommendations for the Handling of Laboratory Animals for Biomedical Research compiled by the Committee on Safety and Ethical Handling Regulations for Laboratory Animals Experiments, Kinki University. The ethical procedures followed and met the requirements of the United Kingdom Coordinating Committee on Cancer Research guidelines. To evaluate tumorigenicity, a suspension of 5 × 10^6^ Sui66- and Sui68-transfectant cells (in 50 μL PBS) were subcutaneously inoculated into both flanks of nude mice (n = 7). To evaluate the tumor growth, a suspension of 5 × 10^6^ Sui68- and Sui73-transfectant cells (in 50 μL PBS) with 50 μL of Matrigel were subcutaneously inoculated into the right flanks of nude mice (n = 5). The tumor volume was calculated as the length × width^2^ × 0.5. The tumor formation and volume were assessed every 2 to 3 days. At the end of the experiment, the mice were sacrificed and the xenografts were resected, fixed in 10% buffered formalin for 6 to 10 hours, and processed for histologic analysis. The method was described previously [38].

### Patients and samples

A total of 29 patients who had been diagnosed as having unresectable PC based on the results of an endoscopic biopsy performed at Kinki University Hospital between April 2007 and March 2008 were enrolled. This study was retrospectively performed and was approved by the institutional review board of the Kinki University Faculty of Medicine. The staging of the PC was determined according to the TNM classification. Among those who had a good performance status and received chemotherapy, the PFS was defined as the time from the initiation of chemotherapy until the first observation of disease progression or death from any cause, OS was defined as the time from the initiation of chemotherapy until death from any cause. The response to chemotherapy was evaluated at one month after the start of therapy and every 2 months thereafter using computed tomography according to the Response Evaluation Criteria in Solid Tumors.

### DNA extraction

The endoscopic biopsy samples were immediately stored at −80°C. Other biopsy samples obtained from the same location were reviewed by a pathologist to confirm the presence of tumor cells. The DNA was extracted using a QIAamp DNA Micro kit (Qiagen, Hilden, Germany) as described previously [36]. The DNA concentration was determined using the NanoDrop2000 (Thermo Fisher Scientific, Waltham, MA).

### Statistical analysis

Continuous variables were analyzed using the Student *t*-test, and the results were expressed as the average and standard deviations (SD). Dichotomous variables were analyzed using the Fisher exact test. PFS and OS were analyzed using the Kaplan-Meier method and were compared among groups using the log-rank test. The statistical analyses were two-tailed and were performed using Microsoft Excel (Microsoft, Redmond, WA). A *P*-value of less than 0.05 was considered statistically significant.

## Abbreviations

ACVR: Activin A receptor; CGH: Comparative genomic hybridization; OS: Overall survival; PBS: Phosphate-buffered saline; PC: Pancreatic cancer; PFS: Progression-free survival; RT-PCR: Reverse-transcription PCR; Scr: Scramble; SD: Standard deviations; sh: Short hairpin; TGFB: Transforming growth factor beta; TGFBR: Transforming growth factor beta receptor.

## Competing interests

The authors declare that they have no competing interests.

## Authors’ contributions

YT designed and participated in the experiments and drafted the manuscript. HS and MK collected clinical samples. HH and MT carried out the experiments with cells. VM and YF carried out the *in vivo* experiments. YK and KS carried out the clinical sample analyses. AI reviewed the histology. ST performed the statistical analysis. MK and KN conceived the study, participated in its design and coordination, and helped to draft the manuscript. All the authors have read and approved the final manuscript.

## Supplementary Material

Additional file 1 **Effects of ligands on the Sui70 cell line (wild-type *ACVR1B* gene and homozygous deletion of *SMAD4* gene).** A. Influence of TGFB1 and activin A on cellular growth in the Sui70 cell line. Both TGFB1 (0.1 ng/mL, *P* = 0.42; 1 ng/mL, *P* = 0.65; 10 ng/mL, *P* = 0.30) and activin A (1 ng/mL, *P* = 0.38; 10 ng/mL, *P* = 0.47; 100 ng/mL, *P* = 0.35) did not influence the cellular growth. hDEL, homozygous deletion; WT, wild-type. B. Western blot analyses for non-SMAD pathway and p21. Both AKT and ERK1/2 had already been phosphorylated, and the phosphorylation was not enhanced by activin A. The expression of p21 was not changed by activin A. Click here for file
