# Supplementary material for: Homozygous deletion of the activin A receptor, type IB gene is associated with an aggressive cancer phenotype in pancreatic cancer
Source: Mol Cancer. 2014 May 27;13:126. doi: 10.1186/1476-4598-13-126 (PMC4047430; doi:10.1186/1476-4598-13-126)
Supplement: Additional file 1 — Effects of ligands on the Sui70 cell line (wild-type ACVR1B gene and homozygous deletion of SMAD4 gene). A. Influence of TGFB1 and activin A on cellular growth in the Sui70 cell line. Both TGFB1 (0.1 ng/mL, P = 0.42; 1 ng/mL, P = 0.65; 10 ng/mL, P = 0.30) and activin A (1 ng/mL, P = 0.38; 10 ng/mL, P = 0.47; 100 ng/mL, P = 0.35) did not influence the cellular growth. hDEL, homozygous deletion; WT, wild-type. B. Western blot analyses for non-SMAD pathway and p21. Both AKT and ERK1/2 had already been phosphorylated, and the phosphorylation was not enhanced by activin A. The expression of p21 was not changed by activin A. [file 1476-4598-13-126-S1.pdf]

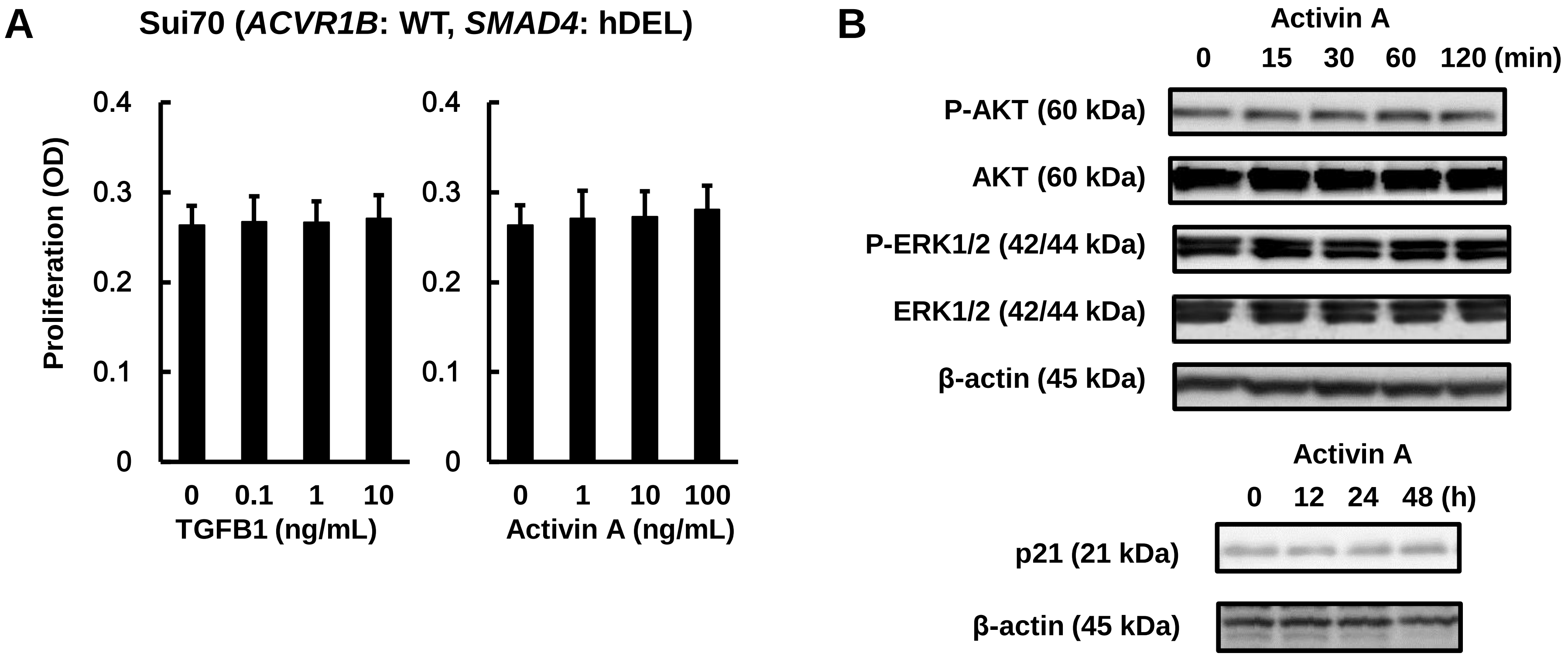

Effects of ligands on the Sui70 cell line (wild-type *ACVR1B* gene and homozygous deletion of *SMAD4* gene). A. Influence of TGFB1 and activin A on cellular growth in the Sui70 cell line. Both TGFB1 (0.1 ng/mL, P = 0.42; 1 ng/mL, P = 0.65; 10 ng/mL, P = 0.30) and activin A (1 ng/mL, P = 0.38; 10 ng/mL, P = 0.47; 100 ng/mL, P = 0.35) did not influence the cellular growth. hDEL, homozygous deletion; WT, wild-type. B. Western blot analyses for non-SMAD pathway and p21. Both AKT and ERK1/2 had already been phosphorylated, and the phosphorylation was not enhanced by activin A. The expression of p21 was not changed by activin A.
